# Histone deacetylase inhibitors suppress *ACE2* and *ABO* simultaneously, suggesting a preventive potential against COVID-19

**DOI:** 10.1038/s41598-021-82970-2

**Published:** 2021-02-09

**Authors:** Yoichiro Takahashi, Akira Hayakawa, Rie Sano, Haruki Fukuda, Megumi Harada, Rieko Kubo, Takafumi Okawa, Yoshihiko Kominato

**Affiliations:** Department of Legal Medicine, Graduate School of Medicine, Gunma University, Maebashi, 371-8511 Japan

**Keywords:** Risk factors, Haematological diseases, Viral infection, Drug discovery

## Abstract

Coronavirus disease 2019 (COVID-19) caused by severe acute respiratory syndrome coronavirus 2 (SARS-CoV-2) has spread worldwide as a pandemic throughout 2020. Since the virus uses angiotensin-converting enzyme 2 (ACE2) as a receptor for cellular entry, increment of ACE2 would lead to an increased risk of SARS-CoV-2 infection. At the same time, an association of the ABO blood group system with COVID-19 has also been highlighted: there is increasing evidence to suggest that non-O individuals are at higher risk of severe COVID-19 than O individuals. These findings imply that simultaneous suppression of *ACE2* and *ABO* would be a promising approach for prevention or treatment of COVID-19. Notably, we have previously clarified that histone deacetylase inhibitors (HDACIs) are able to suppress *ABO* expression in vitro. Against this background, we further evaluated the effect of HDACIs on cultured epithelial cell lines, and found that HDACIs suppress both *ACE2* and *ABO* expression simultaneously. Furthermore, the amount of ACE2 protein was shown to be decreased by one of the clinically-used HDACIs, panobinostat, which has been reported to reduce B-antigens on cell surfaces. On the basis of these findings, we conclude that panobinostat could have the potential to serve as a preventive drug against COVID-19.

## Introduction

Since the first patient was recognized in December 2019, coronavirus disease 2019 (COVID-19) has become a worldwide pandemic^1^, with more than 58.9 million cases and 1.3 million deaths as of late November 2020, according to the WHO online dashboard (https://covid19.who.int/). The disease is caused by novel severe acute respiratory syndrome coronavirus 2 (SARS-CoV-2), which uses angiotensin-converting enzyme 2 (ACE2) as a receptor for cellular entry^2,3^. After binding of the SARS-CoV-2 spike (S) protein to ACE2, host proteases, principally transmembrane serine protease 2 (TMPRSS2), promote cellular entry of the virus^3,4^. These events are likely to occur in specific subsets of epithelial cells of the respiratory and gastrointestinal tracts, which express both *ACE2* and *TMPRSS2*^4,5^. Interestingly, *ACE2* is suggested to be an interferon-stimulated gene and thus upregulated during inflammation, resulting in enhanced SARS-CoV-2 infection^4^. In addition, ACE2 is also considered to affect the pathophysiological process of multiple organ damage including acute lung injury^6^. These findings imply that increased expression of ACE2 would increase the risk of COVID-19^7,8^, whereas reduction of ACE2 might be a promising therapeutic approach for COVID-19^9,10^. However, no established method for reduction of ACE2 to prevent or relieve COVID-19 has been reported.

One of the other factors related to the risk of COVID-19 is the ABO blood group system^11,12^. The ABO system is composed of complex carbohydrate structures that are biosynthesized by A and B transferase encoded by the *A* and *B* alleles on the *ABO* gene, respectively^13^. While A- or B-antigens were originally identified on human red blood cells, they can also be expressed on epithelial cells of the respiratory and gastrointestinal tracts^14^. Although the precise mechanisms are still being investigated, accumulating reports suggest that individuals with the A blood group type are at increased risk for symptoms related to SARS-CoV-2 infection, such as acute respiratory syndrome and cardiovascular diseases, as well as severe outcomes including intubation and death^15–17^. Furthermore, a recent genome-wide association study has clarified that the 9q34.2 locus, which coincides with the *ABO* locus, is one of the two loci that are most significantly associated with severe COVID-19 with respiratory failure, representing a higher risk for blood group A individuals^18^. Studies so far suggest that O individuals are at lower risk from COVID-19 than non-O individuals. Taken together, these findings suggest that the ABO system could be another druggable target for alleviation of COVID-19 risk, i.e. reduction of A- and B-antigens might reduce the risks of COVID-19.

Previously, we had clarified that clinically used histone deacetylase inhibitors (HDACIs) suppress *ABO* expression in vitro, leading to a decrease of B-antigens on the surface of KATOIII cells, a gastric cancer cell line^19^. Therefore, we hypothesized that HDACIs could potentially serve as drugs to prevent severe outcomes of COVID-19. Against this background, in the present study, we further investigated the effect of HDACIs on the expression of *ABO*, as well as that of *ACE2* and *TMPRSS2*, in cultured epithelial cell lines, to determine whether HDACIs could have a potential preventive effect against COVID-19. Given the fact that development of novel vaccines for SARS-CoV-2 would likely take considerable time^20,21^, our findings suggest that HDACIs would warrant clinical investigation to further evaluate their potential usage in this context.

## Results

### HDACIs such as sodium butyrate and panobinostat suppress *ACE2* expression in KATOIII cells

**Figure 1:**
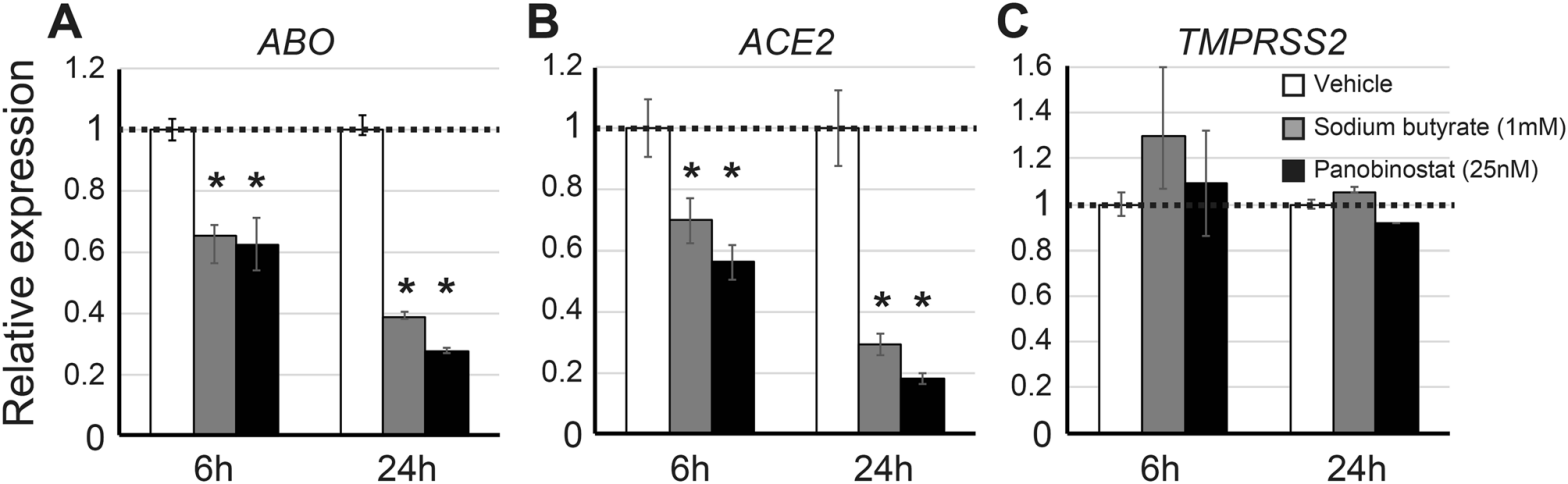
*ABO*, *ACE2* and *TMPRSS2* expression in KATOIII cells cultured with or without sodium butyrate or panobinostat for 6 or 24 h. (**A**–**C**) Relative amounts of *ABO* (**A**), *ACE2* (**B**) and *TMPRSS2* (**C**) transcripts in KATOIII cells treated with or without HDACIs such as sodium butyrate and panobinostat. Clear bars indicate the basal expression levels in the absence of HDACIs, the gray bars represent the relative amounts of transcripts in the presence of 1 mM sodium butyrate, and the solid bars denote those in the presence of 25 nM panobinostat. The graphs express mean fold values relative to those without HDACI. In each panel, the left-hand bars show the results obtained 6 h after incubation with or without HDACIs, while the right-hand bars show data 24 h after treatment. The asterisks represent a significant reduction compared to the values without HDACI (*p* < 0.05).

We have previously reported that HDACIs such as sodium butyrate and panobinostat suppress *ABO* expression in the gastric cancer cell line KATOIII^19^. To examine whether the HDACIs also decrease *ACE2* expression, we performed quantitative real-time PCR (qPCR) on KATOII cells treated with or without 1 mM sodium butyrate or 25 nM panobinostat for 6 or 24 h, targeting *ABO*, *ACE2* and *TMPRSS2* transcripts as well as *β-actin* (*ACTB*) as endogenous control. As we had shown before, these HDACIs suppressed *ABO* expression (Fig. 1A). In addition, it was also clarified that they suppressed the expression of *ACE2* in a time-dependent manner (Fig. 1B). On the other hand, the HDACIs did not suppress *TMPRSS2* (Fig. 1C), suggesting that the HDACI-related suppression was gene-specific.

### Various HDACIs suppress *ACE2* expression in KATOIII cells

**Figure 2:**
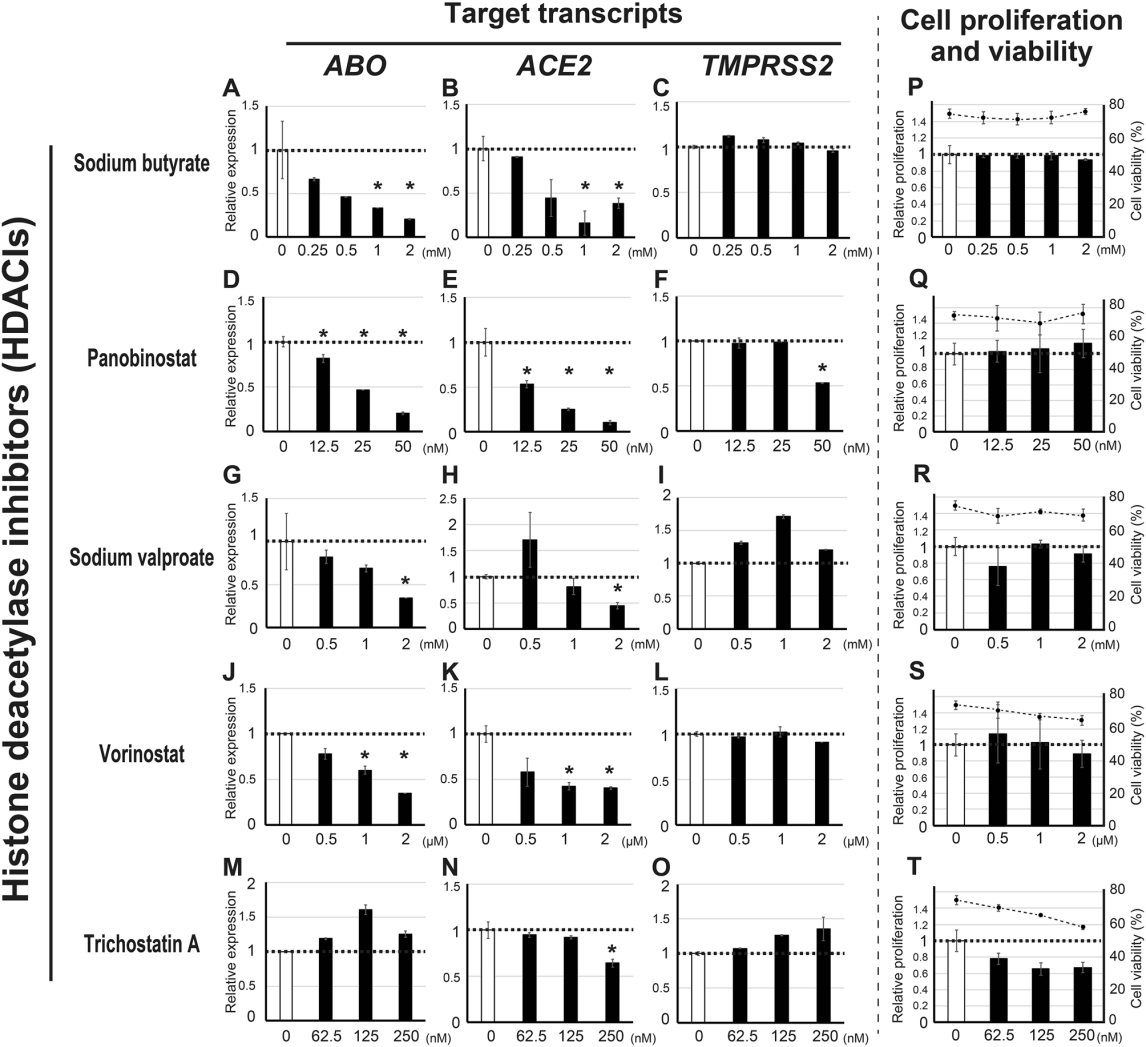
Alterations in the expression of *ABO*, *ACE2* and *TMPRSS2*, cell proliferation, and viability by treatment of KATOIII cells with HDACIs. (**A**–**O**) Each panel represents the relative amount of *ABO* (**A**, **D**, **G**, **J** and **M**), *ACE2* (**B**, **E**, **H**, **K** and **N**) or *TMPRSS2* (**C**, **F**, **I**, **L** and **O**) transcripts in KATOIII cells incubated with or without various concentrations of sodium butyrate (**A**–**C**), panobinostat (**D**–**F**), sodium valproate (**G**–**I**), vorinostat (**J**–**L**) or trichostatin A (**M**–**O**) for 24 h. The amounts are expressed as mean fold values relative to those without HDACI. The asterisks represent a significant reduction compared to the values without HDACI (*p* < 0.05). (**P**–**T**) Each panel represents both the proliferation and viability of KATOIII cells incubated with or without various concentrations of HDACIs for 24 h. The bars indicate cell proliferation as the number of live cells relative to that without HDACI treatment on the left y-axes, while the dots and lines denote the absolute cell viability on the right y-axes.

To further evaluate the effect of HDACIs on *ABO*, *ACE2* and *TMPRSS2* expression, KATOIII cells were treated with several concentrations of various HDACIs including sodium valproate, vorinostat and trichostatin A for 24 h, and the relative amounts of these transcripts were evaluated by qPCR (Fig. 2A–O). In addition, the cell proliferations and viabilities were evaluated in each condition to reveal potential cytotoxicity of the HDACIs (Fig. 2P–T). As expected, sodium butyrate and panobinostat reduced the amount of both *ABO* and *ACE2* transcripts simultaneously in a dose-dependent manner. In addition, sodium valproate and vorinostat caused similar suppression of both *ABO* and *ACE2*, except that a low concentration (0.5 mM) of sodium valproate increased the amount of *ACE2* (Fig. 2H). On the other hand, trichostatin A rarely reduced the *ABO* and *ACE2* transcripts (Fig. 2M,N), while moderately reducing the cell proliferations and viabilities (Fig. 2T). Those observations suggested the considerable cytotoxicity of trichostatin A. None of the HDACIs had a suppressive effect on *TMPRSS2*, except for a high concentration (50 nM) of panobinostat, which suppressed *TMPRSS2* (Fig. 2F). On this basis, we concluded that the HDACIs used in the present study, except for trichostatin A, had the potential to suppress *ABO* and *ACE2* concurrently on KATOIII cells, whereas such suppression was rarely observed for the *TMPRSS2* transcript.

### Suppression of *ABO* and *ACE2* by HDACIs on other cell lines

**Figure 3:**
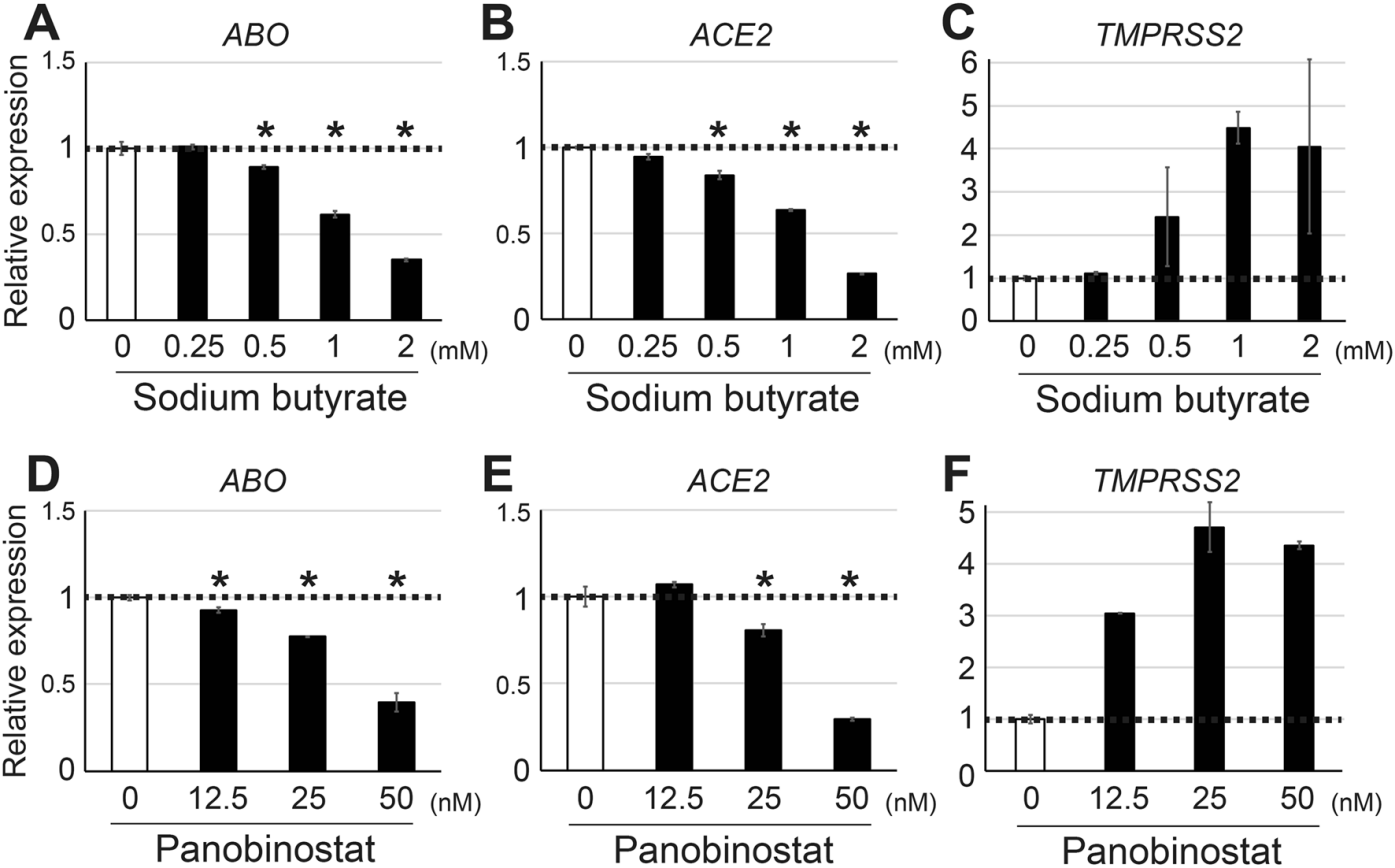
Suppression of *ABO* and *ACE2* in NUGC-4 cells treated with sodium butyrate or panobinostat for 24 h. (**A**–**C**) Relative amounts of *ABO* (**A**), *ACE2* (**B**) and *TMPRSS2* (**C**) transcripts in NUGC-4 cells treated with various concentrations of sodium butyrate for 24 h. (**D**–**F**) Relative amounts of *ABO* (**D**), *ACE2* (**E**) and *TMPRSS2* (**F**) transcripts in NUGC-4 cells treated with various concentrations of panobinostat for 24 h. The transcript amounts are expressed as mean fold values relative to those without HDACI. The asterisks represent a significant reduction compared to the untreated values (*p* < 0.05).

Next, we sought to evaluate the HDACI-related suppression of *ABO* and *ACE2* on other cell lines. We first quantified the basal expression of *ABO*, *ACE2* and *TMPRSS2* on several cell lines that were available in our laboratory (Table 1). As a result, we found that, in addition to KATOIII cells, the gastric cancer cell line NUGC-4 also expressed considerable amounts of *ABO*, *ACE2* and *TMPRSS2* transcripts, while all the other cell lines including a lung derived cell line, HMVEC–L, expressed an insufficient amount of at least one of the three transcripts. Therefore, the NUGC-4 cell line was deemed relevant for further evaluation of the HDACI-related suppression of *ABO* and *ACE2*. Against this background, we performed similar qPCR experiments on NUGC-4 cells incubated with or without various concentrations of sodium butyrate or panobinostat (Fig. 3), resulting in similar suppression of *ABO* and *ACE2* by the HDACIs, while *TMPRSS2* was not suppressed. Thus, it was clarified that HDACIs such as sodium butyrate and panobinostat had the ability to suppress both *ABO* and *ACE2* simultaneously in several epithelial cell lines.

**Table 1:** Expression levels of *ABO*, *ACE2* and *TMPRESS2* in various cultured cell lines.

Cell line	Characteristics	Expression of transcripts^a^		
		*ABO*	*ACE2*	*TMPRSS2*
KATOIII	Gastric adenocarcinoma	1530	218	15292
NUGC-4	Gastric adenocarcinoma	2341	1107	2771
MKN1	Gastric adenosquamous carcinoma	54	6	101
SW480	Colon adenocarcinoma	14	Less than 1	149
SV-HUC	Uroepithelium cell	59	2	174
5637	Bladder carcinoma	229	18	1886
KK47	Bladder carcinoma	Less than 1	Less than 1	Less than 1
T24	Bladder carcinoma	Less than 1	Less than 1	94
HMVEC-L	Human lung microvascular endothelial cell	Less than 1	Less than 1	Less than 1
SH-SY5Y	Neuroblastoma	Less than 1	5	14
LAN-5	Neuroblastoma	Less than 1	3	Less than 1
K562	Erythroleukemic cell	2359	5	6

^a^Expression levels are presented as means of duplicate determinations for copy numbers of target transcript per 10^4^ copy numbers of *ACTB* transcript.

### HDACIs decrease the ACE2 protein in cell lysates of KATOIII and NUGC-4

Whether or not the HDACI-related suppression of *ACE2* would lead to a reduced amount of ACE2 protein in cultured cell lines was considered an intriguing issue. Notably, we had shown previously that panobinostat reduced the amount of B-antigens on KATOIII cells. Therefore, among the various HDACIs, we decided to focus on panobinostat, considering that it might serve as a preventive drug against COVID-19 by simultaneously diminishing A- or B-antigens and ACE2 proteins on the cell surface.

**Figure 4:**
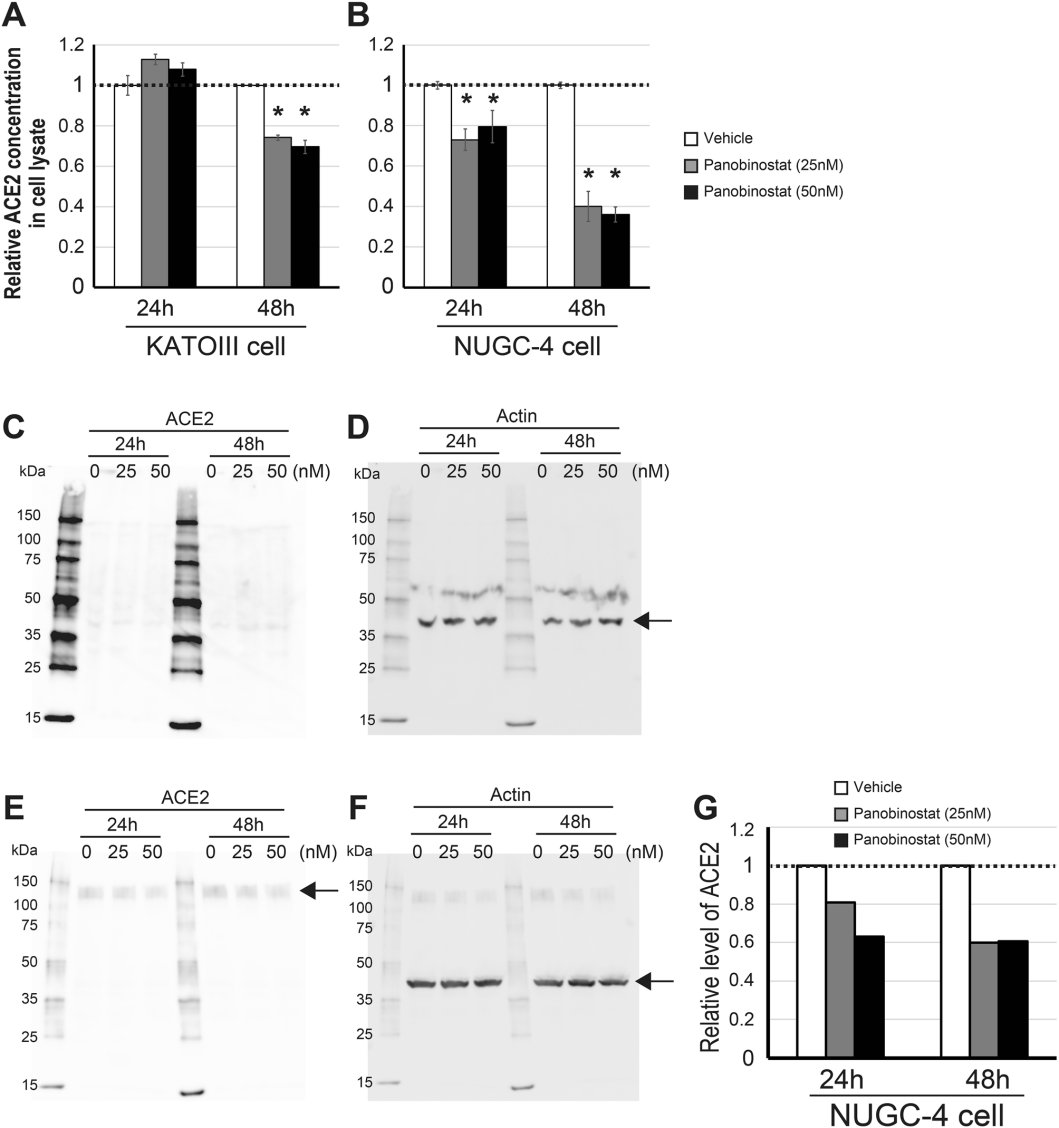
Reduction of the ACE2 amount in KATOIII and NUGC-4 cells treated with panobinostat. (**A**, **B**) Relative amounts of ACE2 in KATOIII (**A**) or NUGC-4 (**B**) cells incubated with 25 nM or 50 nM panobinostat for 24 or 48 h, analyzed by ELISA. The clear bars indicate the basal amount of ACE2 in the absence of panobinostat, the gray bars represent the relative amounts the ACE2 in the presence of 25 nM panobinostat, and the solid bars denote those in the presence of 50 nM panobinostat. The graphs express the mean fold values relative to those without HDACI. In each panel, the left-hand bars show the results obtained 24 h after incubation with or without panobinostat, while the right-hand bars show the data 48 h after treatment. The asterisks represent a significant reduction compared to the untreated values (*p* < 0.05). (**C**–**F**) Western blots for cell lysates derived from KATOIII (**C**, **D**) and NUGC-4 (**E**, **F**) cells treated with 0, 25 or 50 nM panobinostat for 24 or 48 h. Panels (**C**) and (**E**) represent ACE2 while panels (**D**) and (**F**) indicate actin as a loading control. The amount of protein applied to each lane was 100 or 40 μg for the KATOIII or NUGC-4 assays, respectively. In panel (**C**), no ACE2 is illustrated because of its low expression. The arrows in the panels (**D**) and (**F**) indicate actin with a band size of 42 kDa. The arrow in panel (**E**) represents ACE2 with a band size of 120 kDa. (**G**) Relative densitometric levels of ACE2 in NUGC-4 lysates divided by the amounts of actin. The clear bars indicate the basal amount of ACE2 in the absence of panobinostat, the gray bars represent the relative amounts of ACE2 in the presence of 25 nM panobinostat, and the solid bars denote those in the presence of 50 nM panobinostat. The left-hand bars show the results obtained 24 h after incubation with or without panobinostat, while the right-hand bars show the data 48 h after treatment.

To this end, we performed enzyme-linked immunosorbent assays (ELISA) using cell lysates prepared from KATOIII and NUGC-4 cells incubated with 0, 25 or 50 nM panobinostat for 24 or 48 h (Fig. 4). For KATOIII cells, we confirmed that the amount of ACE2 was reduced at 48 h after incubation with 25 nM and 50 nM panobinostat (Fig. 4A). For NUGC-4 cells, ACE2 reduction was observed after both 24- and 48-h treatment with 25 nM and 50 nM panobinostat (Fig. 4B). There was no significant difference in the amount of ACE2 between the two concentrations of panobinostat. Furthermore, we carried out additional Western blot analysis of whole-cell lysates prepared from KATOIII and NUGC-4 cells cultured under the same conditions as those for the above ELISA. For KATOIII cells, however, we rarely detected ACE2 in the lysates even though no panobinostat was added (Fig. 4C,D), probably because of the relatively low level of *ACE2* expression in KATOIII cells (Table 1). On the other hand, for NUGC-4 cells, it was clarified that panobinostat at both 25 nM and 50 nM decreased the amount of ACE2 after treatment for both 24 and 48 h (Fig. 4E–G). In conclusion, panobinostat was able to reduce the amount of ACE2 in cultured epithelial cells.

## Discussion

We revealed that several HDACIs suppressed *ABO* and *ACE2* transcripts concurrently in KATOIII or NUGC-4 cells, while *TMPRSS2* expression was rarely repressed. Notably, among the HDACIs used in the present study, panobinostat caused drastic suppression at the lowest concentrations, whereas trichostatin A barely reduced those transcripts probably because of its cytotoxicity. Finally, panobinostat reduced the amount of ACE2 proteins in both KATOIII and NUGC-4 cells. Considering together the findings that panobinostat decreases B-antigen on the KATOIII cells^19^, that non-O individuals have a higher risk of COVID-19^18^ and that higher expression of ACE2 is a risk factor for COVID-19^7–10^, it seems plausible that panobinostat could have the potential to serve as a preventive drug against COVID-19.

Currently, the association of panobinostat with *ACE2* expression is also being investigated by two other research groups^22,23^. Those two groups adopted very similar approaches; they re-analyzed the same publically available data, comprising gene expression profiles for thousands of perturbagens at a variety of time points, doses, and cell lines^24^ in order to identify drugs that could significantly modify *ACE2* expression. Although their interpretations of the results differed, to our surprise, both of their analyses suggested that panobinostat might up-regulate *ACE2* expression, contrary to our findings. Although the reason for this contradiction is unclear, we speculate that it might be attributable to differences in experimental conditions between our data and those publically available. For example, the former group, He and Garmire, analyzed expression profiles in the presence of 10 µM panobinostat, which was more than 200 times higher than the concentration we employed^22^. In addition, neither KATOIII nor NUGC-4 were featured in the publically available data^24^. As shown in Table 1, few cell lines seem to express a sufficient amount of *ACE2*, and thus most cultured cells are unsuitable for investigating regulation of the gene^4,25^. Therefore, the effects of HDACIs on gene expression in vitro need to be evaluated carefully. On the other hand, Xu et al. recently reported a novel approach whereby conventional molecular docking was computationally accelerated in combination with generative artificial intelligence, resulting in the identification of potential drug-repurposing candidates for COVID-19^26^. Surprisingly, though their approach was completely different from ours, panobinostat was identified as one of the six candidate drugs. Considering this accumulating evidence to suggest the preventive potential of panobinostat against COVID-19, further evaluations, including clinical trials, seem warranted. Notably, the in vitro potencies of valproic acid, panobinostat and vorinostat as therapeutics for various cancers were reported to be in the mM, µM and nM order, respectively^27^, whereas our data revealed that panobinostat might have preventive potential against COVID-19 at concentrations as low as the nM order. Although future in vivo or clinical studies will be needed to clarify the possible tolerable doses of HDACIs for potential prevention of COVID-19, our present data suggest that panobinostat in particular could be employed clinically at especially low doses, leading to less adverse effects.

In some experiments, we observed slight up-regulation of *TMPRSS2* in KATOIII cells when the cells were treated with 1 mM sodium butyrate for 6 h (Fig. 1C) or 1 mM sodium valproate for 24 h (Fig. 2I). In addition, the NUGC-4 cells showed an apparent increase in *TMPRSS2* expression in the presence of HDACIs such as sodium butyrate and panobinostat (Fig. 3C,F). While these results suggest potential activation of the host protease TMPRSS2 by HDACIs, this might be irrelevant to the risk of viral infection considering that TMPRSS2 is involved in the process of viral entry only after the virus has bound to ACE2 on epithelial cells^3,4^. Whether HDACIs actually inhibit the cellular entry of the virus will need to be clarified by future experiments using live virus.

An increasing number of reports have suggested an association between the ABO system and COVID-19^11,12,14–18^. This is not surprising, since involvement of the ABO system in viral infection, including SARS, has been documented^28^, although the precise mechanism remains unclear. It is controversial whether the association of the ABO system with COVID-19 could be attributable to the amount of A- or B-antigens on cells or anti-A or -B antibodies in serum^11,12,14^. Interestingly, Ladikou et al. have reported that patients with severe COVID-19 who developed venous thromboembolism had highly elevated levels of von Willebrand factor (vWF) and coagulation factor VIIIc^29^, whose serum levels are correlated with the ABO system and are higher in non-O individuals^30,31^. Considering that the presence of A- or B-antigens in vWF N-linked oligosaccharides plays a role in vWF levels^32,33^, reduction of the A- or B-antigen might be a reasonable approach for reducing plasma vWF levels as well as the risk of thrombopoietic symptoms of COVID-19. Notably, the ABO system is also associated with a number of other factors, including ACE plasma activity^34^ and interleukin levels^35,36^. Thus, the influence of the ABO system seems to be complex, highly diversified and much more significant than has been clarified^33^. Further investigations focusing on the ABO blood group system should help to reveal the hidden roles of this system that could significantly impact human health, disease and biology.

## Materials and methods

### Cell culture with or without HDACIs

The cell lines KATOIII (JCRB0611), K562 (JCRB0019), NUGC-4 (JCRB0834) and MKN1 (JCRB0252) were originally purchased from Japanese Collection of Research Bioresources Cell Bank (Osaka, Japan), SW480 (ATCC CCL-228), SV-HUC (ATCC CRL-9520), HMVEC-L (ATCC CC-2527) and SH-SY5Y (ATCC CRL-2266) were from American Type Culture Collection (Manassas, VA), 5637 (TKG 0605), KK47 (TKG 0663) and T24 (TKG 0443) were from Cell Resource Center for Biomedical Research (Miyagi, Japan) and LAN-5 (RCB0485) was from RIKEN BioResource Research Center (Ibaraki, Japan). The KATOIII and K562 cells were cultured as described previously^19^. The NUGC-4, MKN1, SW480, 5637, KK47 and LAN-5 cell lines were cultured in RPMI1640 medium containing 10% FCS, 100 U/ml penicillin and 100 µg/ml streptomycin. The culture medium for SV-HUC, T24, HMVEC-L or SH-SY5Y was Ham’s F12K, MEM, EGM-2MV Microvascular Endothelial Cell Growth Medium-2 or 1:1 mixture of MEM and F12, each with 10% FCS, 100 U/ml penicillin and 100 µg/ml streptomycin. For treatment with the HDACIs, the cells were seeded at a density of 2.5 × 10^5^/ml one day before the experiment. On the following day, the cells were re-seeded at a density of 2.5 × 10^5^/ml in new medium with or without HDACIs. The medium was not changed thereafter until harvest of the cells. The HDMCIs we used included sodium butyrate (#303410; Sigma-Akdrich), panobinostat (#13280; Cayman Chemical Company), sodium valproate (#13033; Cayman Chemical Company), vorinostat (#10009929; Cayman Chemical Company) and trichostatin A (#89730; Cayman Chemical Company). The solvent used for sodium butyrate and sodium valproate was deionized-distilled water, and that used for panobinostat, vorinostat and trichostatin A was dimethyl sulfoxide.

KATOIII cell proliferation and viability were evaluated by both manual and automatic counting of live or dead cells after trypan blue staining in each condition where the cells were treated with various concentrations of each HDACI for 24 h. The automatic counting was performed with a Countess II FL Automated Cell Counter (#AMQAF1000, Invitrogen). Every count was conducted at least twice.

### Quantitative real-time PCR (qPCR)

RNA purification, cDNA preparation and quantification of *ABO* and *ACTB* transcripts were performed as described previously^19^. qPCR of the *ACE2* and *TMPRSS2* transcripts was performed with the specific primer sets “ACE2 Primer 2” and “TMPRSS2 Primer 2”, respectively, as described by Ma et al*.*^37^, under the following conditions: 95 °C for 3 min and 40 cycles at 95 °C for 3 s and at 60 °C for 30 s. Every assay was conducted at least twice, and the absolute amount of each transcript determined by qPCR was standardized by the amount of *ACTB* transcript.

### Enzyme-linked immunosorbent assay (ELISA)

ELISA was performed using a Human ACE2 ELISA Kit (#ab235649, Abcam) following manufacturer’s instructions. Briefly, KAKTOIII or NUGC-4 cells were harvested 24 or 48 h after incubation with or without HDACIs, and solubilized in 1 × cell extraction buffer PTR. After centrifugation, the concentration of total protein in the supernatant was measured using a DC protein assay kit (#5000112JA, Bio-Rad), and 250 or 100 ng of total protein derived from KATOIII or NUGC-4 cell lysates, respectively, was applied to each well of a ready-to-use microplate provided in the kit. Then the ACE2 antibody cocktail was added to each well, followed by 1-h incubation at RT on a plate shaker set to 400 rpm. After the incubation, each well was rinsed three times, TMB Development Solution was added, and incubation was performed at RT on a plate shaker. Ten minutes later, the Stop Solution was added to each well, and the end point reading of OD at 450 nm was recorded using a iMark microplate absorbance reader (#168-1135, Bio-Rad). The concentration of the ACE2 protein in the sample was determined by interpolating the blank control subtracted absorbance values against the standard curve. Every assay was conducted in duplicate.

### Western blotting

Whole-cell lysates were prepared from KATOIII and NUGC-4 cells incubated with 0, 25 or 50 nM panobinostat for 24 or 48 h. One hundred or 40 µg of total protein was applied to each lane for the KATOIII or NUGC-4 assays, respectively. The transferred membrane was reacted with a recombinant rabbit anti-ACE2 monoclonal antibody (#ab108252, Abcam), followed by treatment with anti-rabbit immunoglobulins/HRP (#P0448, DakoCytomation) and Amersham ECL Prime Western Blotting Detection Reagent (#RPM2232, GE Healthcare). Then, densitometry measurements were conducted with a LAS-3000 and MultiGauge v3.0 (FujiFilm, Tokyo, Japan). Subsequently, the same membrane was rinsed, processed with blocking buffer, reacted with mouse anti-β-actin monoclonal antibody (#017-24551, Wako) and anti-mouse IgG (H + L)-HRP conjugate (#172-1011, BioRad). Detection and densitometry measurements were performed again as described above.
